# A Study of the Central Motor Drives Interactions Between the Eyes, and an Index Finger, and a Little Finger

**DOI:** 10.3390/brainsci15040422

**Published:** 2025-04-20

**Authors:** Shiho Fukuda, Han Gao, Naoki Hamada, Koichi Hiraoka

**Affiliations:** 1 School of Rehabilitation Science, Graduate School of Osaka Metropolitan University, 3-7-30 Habikino, Habikino 583-8555, Osaka, Japan; vqnw01142@ares.eonet.ne.jp (S.F.); gh1823020@outlook.com (H.G.); w12relax@gmail.com (N.H.); 2 School of Medicine, Osaka Metropolitan University, 3-7-30 Habikino, Habikino 583-8555, Osaka, Japan

**Keywords:** eye–hand coordination, lateral inhibition, surround inhibition, reaction time

## Abstract

**Background/Objectives**: When manipulating an object placed on the palm, the eyes and fingers move together. To perform this task precisely, coordination of the eyes and fingers is needed. Based on this view, the present study examined the three-way interaction among the central motor drives to the eyes, index finger, and little finger. **Methods**: Healthy male participants abducted the right index and/or little finger with or without concomitant saccadic eye movement to the right in response to a visual cue, while the forearm was in the pronated or supinated position. We measured the reaction time (RT), velocity, and amplitude of the eye movements, as well as the RT and amplitude of the electromyographic (EMG) responses in the prime movers for the independent and dependent finger movements. **Results**: The velocity, amplitude, and RT of the eye movement were not changed by the additional involvement of the finger movement, indicating that the central motor drive to the finger does not influence the eye motor excitability and central motor drive to the eyes. On the one hand, the RT of the finger was not changed by the eye movement, indicating that the central motor drive to the eyes does not influence the central motor drive to the finger muscle. On the other hand, the EMG amplitude in the first dorsal interosseous muscle at the movement onset decreased during the concomitant eye movement, indicating that the central motor drive to the eyes suppresses the motor excitability of the independent finger muscle. The RT increased and EMG amplitude decreased in one finger muscle when the other finger concurrently moved, indicating that the central motor drive to one finger muscle suppresses the motor excitability of and central motor drive to the other finger muscle. The change in the RT and EMG amplitude in one finger muscle caused by the concomitant execution of the other finger movement and/or eye movement varied with forearm position, indicating that forearm proprioception influences the interaction of the motor execution processes among the fingers and eyes. **Conclusions**: The central motor drive to the eyes or finger muscles suppresses the motor excitability of the other finger muscles and the central motor drive to that muscle, but the central motor drive to the finger muscles does not influence those for the eyes. Forearm proprioception influences the motor excitability of the finger muscle and central motor drive to that muscle.

## 1. Introduction

When a person manipulates an object held in the palm, they tend to gaze at the moving finger and/or the object. In this case, they move the eyes to keep both the object and fingers at the center of their visual field. To perform this task precisely, two types of interactions are required. One is eye–finger interaction. This interaction has been supported by several previous findings. Velocity changes in the eye and hand movements accompanying the change in the moving target’s direction were similar [1,2]. The posterior parietal cortex was activated not only during the reaching movement but also during the eye movement [3]. During the saccade eye movement accompanied by a pointing movement to a target, the reaction time (RT) of the eye and hand movements was found to be positively correlated [4].

For the eye and finger interaction, two directions of causality must exist: one is from the eyes to the finger, and the other is from the finger to the eyes. First, regarding the causal influence from the eyes to the finger, previous studies reported that the motor execution of the eyes influences the arm or hand motor execution. The saccade eye movement prolonged the RT and decreased the velocity of the concomitantly performed forearm movement [5]. The wrist movement accuracy improved when the eye movement was concomitantly performed [6,7]. The eye movement decreased the duration and increased the accuracy of the arm movement to the target [8].

Second, eye motor execution suppressed corticospinal excitability in finger muscles. The corticospinal excitability in the intrinsic hand muscles at rest was suppressed during the smooth pursuit eye movement [9]. Corticospinal excitability of the first dorsal interosseous muscle (FDI) was facilitated during the smooth pursuit eye movement [10]. The corticospinal excitability of FDI was suppressed during the saccade eye movement with visual occlusion [11]. Taken together, eye motor execution influences the motor process of the arm, hand, and finger.

The central motor drive to the finger may also affect the excitability of the eye motor system. Regarding this issue, only one study has been conducted. The motor plan of the saccade eye movement was suppressed while moving the hand, although this suppression did not occur when the hand was at rest [12]. This suggests that the motor execution of the hand affects the eye movement control. This view may be applicable to the effect of the finger movement on the eye motor control.

The other is the interaction between the motor executions of the fingers. The simple RT of the finger movement was changed by the additional involvement of the other finger movement [13]. The corticospinal excitability in one finger muscle varied according to the involvement of another finger movement [14,15]. The force production of one finger was found to suppress the force production of another finger [16]. The contraction of one finger muscle suppressed the corticospinal excitability of another finger muscle, a phenomenon known as surround inhibition [17,18,19,20,21]. Force production of one finger influenced the force production of the other finger, a phenomenon referred to as the enslaving effect [22,23]. These findings indicate that there is an interaction in motor excitability between the fingers.

Despite these previous findings, there are no studies investigating the three-way interaction of the motor processes among the eyes and fingers. In the present study, three-way interaction between the central motor drives to the eyes and fingers was investigated by examining two mechanisms. One mechanism concerns the intensity of the central motor drive to the eyes or fingers. To investigate this, simple RT was measured. In simple RT, perception of the imperative cue and motor execution are involved [24], but the response selection and cue discrimination are not involved. Thus, simple RT was employed to exclude the additional time required for response selection and cue discrimination. The corticospinal excitability gradually increased in the RT, a phenomenon referred to as pre-movement facilitation [25,26,27,28,29]. The central motor drive is defined as the excitatory descending drive to activate the motor units. Voluntary movement requires central motor drives to be released from the primary motor cortex and transmitted to the spinal motoneurons and effector muscles [30]. Thus, simple RT represents the time required for the central motor drive-induced increase in the motor excitability to reach the threshold. Thus, variations in simple RT reflect the change in the central motor drive-induced increase in the motor excitability required to reach the threshold. In this case, the inhibitory input to the target muscle, induced by the central motor drive to one muscle, causes the increase in the simple RT of the target muscle and vice versa. Thus, we examined whether the central motor drive to the eyes or the finger is affected by the central motor drive to the other by measuring the simple RT.

Another mechanism concerns the recruitment of motor units induced by the central motor drive. The electromyographic (EMG) response is the sum of the action potentials generated by the motor units and represents the amount of the neural activation sent to the muscle [31]. Thus, the EMG amplitude represents the number of the motor units reaching the threshold. In other words, the EMG amplitude is an index of the target muscle’s motor excitability. Based on this view, the alternations in the EMG amplitude indicate the change in the motor excitability resulting from the inhibitory or facilitatory input. Thus, by observing the EMG amplitude, we can infer the inhibitory or facilitatory input to the target muscle.

Somatosensory feedback is delayed by 110–150 ms [32]. Thus, the proprioceptive feedback does not affect the motor process at the movement onset. Based on this view, the EMG amplitude, particularly at the movement onset, reflects the motor excitability without the influence of the afferent feedback. Thus, in the present study, the EMG amplitude at the movement onset was measured to infer the inhibitory or facilitatory input to the target muscle without the influence of the afferent feedback.

The corticospinal excitability of the arm and hand muscles depends on forearm position [33]. This suggests that forearm proprioception influences the motor excitability of the hand muscles. When one gazes at an object on the palm, they maintain the supinated forearm position, but when reaching for an object, they maintain the neutral or pronated forearm position. Thus, forearm proprioception may influence the interaction between the eyes and fingers. Forearm proprioception has been shown to modulate the effect of the central motor drive to the eyes on the corticospinal excitability of the finger muscles [9]. In contrast, such an effect was not observed when the saccade eye movement was performed with visual occlusion [11]. Despite those previous findings, there are no studies that investigated the effect of forearm proprioception on the three-way interaction among the eyes and fingers. The present study examined this issue as well.

Taken together, we investigated whether the motor drive to the eyes influences the motor process underlying finger movements and whether the motor drive to the finger affects the eye movements and the other finger movements. This provides evidence for a three-way interaction among the motor processes of the eyes and fingers. These findings may contribute to the understanding of the coordinative control of the precise finger movements while viewing the moving fingers. In the Discussion section, we propose a simple schematic representation of the hypothesized interactions among the eye, index finger, and little finger movements, based on the present findings.

## 2. Materials and Methods

### 2.1. Participants

Fifteen males aged 35.1 ± 10.0 years participated in this study. Previous studies found gender differences in participants’ physical characteristics [34] and motor performance [35]. Accordingly, we recruited males to avoid the gender-related variability of the motor-related measurements. The sample size was determined by power analysis using G*Power 3.1.9.6 (Heinrich-Heine-University, Duesseldorf, Germany). To achieve 80% power with an alpha of 0.05, twelve participants were required to detect a large effect (f = 0.4) of interaction between the main effects (forearm position and other movements) for two-way repeated measures ANOVA. All of the participants were right-handed, according to the Edinburgh Handedness Inventory [36]. The participants had no history of neurological disease. All participants gave written informed consent for study participation prior to the experiment, and the study design was approved by the ethics committee of Osaka Metropolitan University.

### 2.2. Apparatus

The participants were seated on a chair with their forearms and hands on a table without any physical restriction. The chin was placed on a chin support, and their forehead was kept at a fixed rigid frame to prevent any motion artifact of the head. A white screen with 110 cm height and 150 cm width was presented on a vertical wall 1 m in front of them. A preparation cue on this screen was provided by projection equipment (PJWX4152, RICHO, Tokyo, Japan). Eye movement in the horizontal axis was measured using a cornea reflection device (TK2901, Takei Kiki, Tokyo, Japan). An illuminometer-cum-light sensor, sensitive to the reflection difference between the cornea and sclera, was fixed on a goggle frame. The electrodes recording electrooculographic (EOG) activity of the left orbicularis oculi muscle were placed over the outer and inner corners of the left lower eyelid.

**Figure 1:**
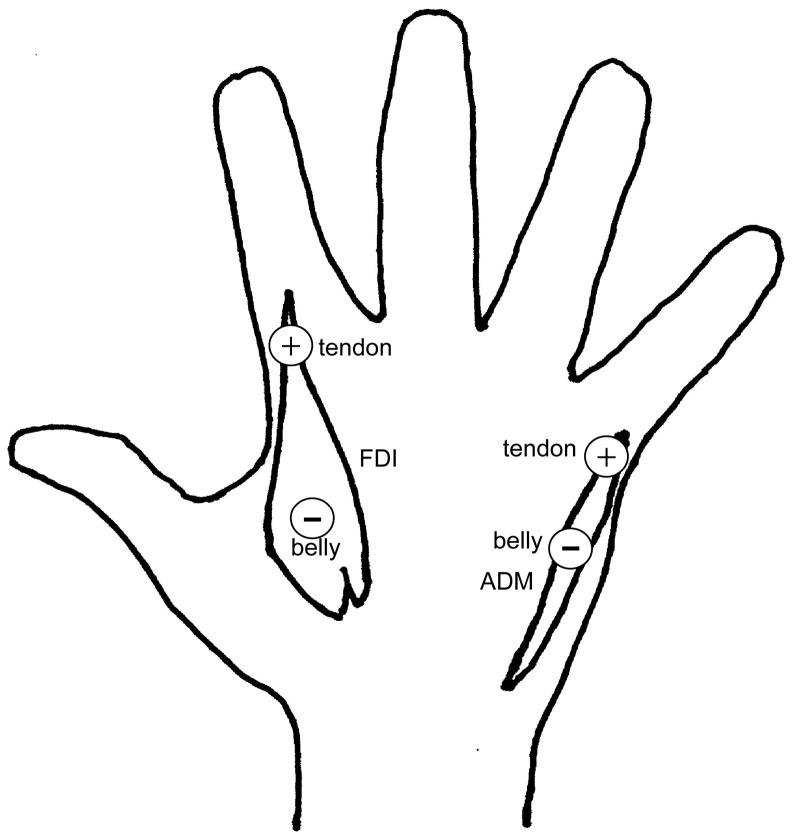
Location of surface recording electrodes of EMG on two muscles.

FDI is the prime mover of the index finger abduction, but the abductor digiti minimi muscle (ADM) is that of the little finger abduction. While the index finger moves independently, the little finger moves with the other fingers [37,38,39,40]. To examine the different roles of the interaction between the independent and dependent fingers, the surface electrodes recording the EMG were placed over the right FDI and ADM with belly-tendon montages (Figure 1). To reduce the impedance level of the electrode sites, an experimenter rubbed the sites with abrasive electrolytic gel.

The EMG and EOG signals were amplified by an amplifier (MEG-2100, Nihon Kohden, Tokyo, Japan). The EMG signals were amplified with a band-pass filter from 15 Hz to 1 kHz. The EOG signals were amplified with a band-pass filter from 0.5 Hz to 1 kHz. The analog signals of the cornea reflection device, EOG, and EMG were converted to digital signals at a sampling rate of 10 kHz using an A/D converter (PowerLab 800S, AD Instruments, Colorado Springs, CO, USA) and were stored in a personal computer. The digital filter (100 Hz high-pass filter) was applied to the EMG traces to remove the motion artifact.

### 2.3. Procedure

The participants maintained the supinated or pronated forearm position on the table. A preparation cue (a circle filled with black) was displayed in the center of the screen, and this cue lasted 3, 4, 5, or 6 s. The participants phasically moved the eyes to the right (E), abducted the index finger (I) or the little finger (L), moved the eyes and index finger together (EI), moved the eyes and little finger together (EL), moved the index and little fingers together (IL), or moved the eyes, index finger, and little finger (EIL) freely without a target in response to the disappearance of the preparation cue. When eye movement was not instructed, they kept their gaze fixed on the screen. An experimenter instructed the participants not to blink while presenting a preparation cue and while moving the eyes and/or fingers to respond to the disappearance of the cue. The experimenter informed the participants of the next task before beginning each trial. The task for each trial was randomly assigned. Ten trials were conducted in each motor task. Because the E, I, L, EI, EL, IL, and EIL tasks were performed in each forearm position, the total number of the task conditions was 14. Taken together, 140 trials were conducted in total. Unsuccessful trials (i.e., trials with eye blinks or incorrect movement) were discarded and retried after completing all other trials. To ensure that participants were familiar with the tasks, practice trials were conducted prior to the test trials.

### 2.4. Data Analysis

**Figure 2:**
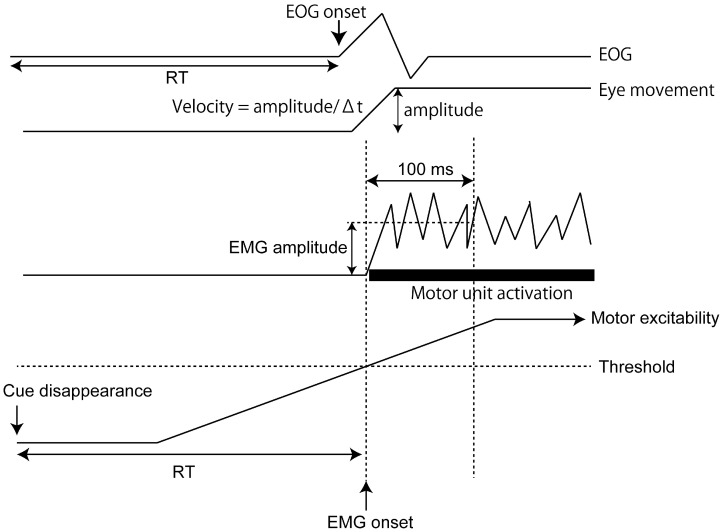
Data analysis of RT and EMG amplitude. EOG, electrooculographic response; EMG, electromyographic response; RT, reaction time.

The EMG traces were fully rectified for the data analysis. The EMG and EOG onset was defined as the time of the earliest rise in the response [41]. The onset of EMG was determined by visual inspection in previous studies [41,42,43,44]. The difference in mean and standard deviation of the response onset when the EMG onset was determined visually and when determined by the automatic detection methods was small [45]. The slope of the regression line between the EMG onset via visual inspection and that via computer-based determination was 0.999 [41]. The intra-rater reliability (test-retest reproducibility within each rater) of the visual determination of the onset of the electromyographic burst was high (ICC3,1 = 0.992) [46]. Accordingly, visual inspection of the EMG onset is as reliable as the algorithm-based determination [41,47]. In a previous study, authors stated that the determination of the EMG onset via algorithm is not sensitive, and thus, visual determination of the EMG onset is recommended [48]. Accordingly, in the present study, the EMG onset, as well as the EOG onset, was visually determined by an experimenter in all trials across the participants. RT was defined as the interval between the time of the disappearance of the preparation cue and EMG or EOG onset. Saccade amplitude was estimated on a base-to-peak basis. The average velocity of the saccade eye movement in the time window between the onset and peak of the eye movement was estimated. The RT of the EOG response was defined as the time between the cue onset and EOG response onset. The RT of the EMG response was defined as the time between the cue onset and the EMG response onset. The rectified EMG amplitude in the time window 0–100 ms after the disappearance of the preparation cue was averaged (Figure 2). The median of the data was used for each task.

Two-way repeated measures ANOVA was conducted for testing the differences in means for two factors [2 (forearm position) ×4 (eye or finger movements)]. The main effect of forearm position was examined to determine whether forearm proprioception influences the motor process. The main effect of the eye or finger movements was examined to determine whether the additional involvement of the central motor command to the eyes or fingers influences the motor process of the other. The result of Greenhouse–Geisser’s correction was reported whenever Mauchly’s test of sphericity was significant. When there was an interaction between the main effects, a test of simple main effect was conducted for each main effect in each level of the other main effect. When ANOVA revealed statistical significance, multiple comparisons between two means were performed using the paired t-test with Bonferroni correction. The alpha level was 0.05 for these statistical analyses. Excel-Toukei 2010 ver. 1.13 (Social Survey Research Information, Tokyo, Japan) was used for statistical analysis. The results are presented as mean and standard error of the mean.

## 3. Results

### 3.1. Eye Movement

**Figure 3:**
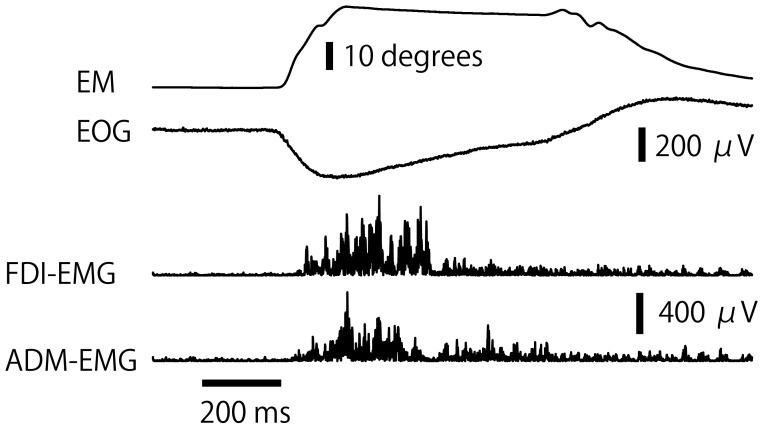
Raw traces of the EOG, eye movement, and EMG. EM, eye movement; EOG, electro-oculography; EMG, electromyography; FDI, first dorsal interosseous muscle; ADM; abductor digiti minimi.

**Figure 4:**
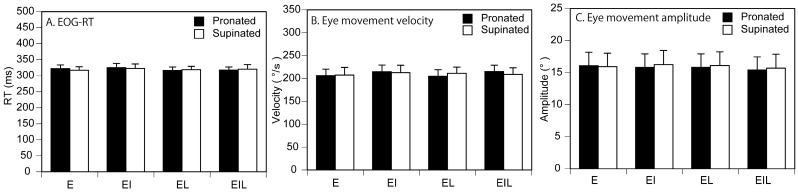
EOG-RT, velocity, and amplitude of eye movements. Data points indicate means and error bars indicate the stand error of the means.

The raw traces of the eye movement, EOG response, and EMG response are shown in Figure 3. The RT of the EOG response is shown in Figure 4A. There was no significant interaction between the main effect of the task and that of forearm position [F (3, 42) = 0.312, *p* = 0.817]. There was no significant main effect of forearm position [F (1, 14) = 0.057, *p* = 0.815] and task [F (3, 42) = 0.715, *p* = 0.549]. The velocity of the saccade eye movement is shown in Figure 4B. There was no significant main effect of either task [F (3, 42) = 1.291, *p* = 0.290] or forearm position [F (1, 14) = 0.000, *p* = 0.989]. There was no significant interaction between the main effect of task and that of forearm position [F (3, 42) = 0.671, *p* = 0.575]. The amplitude of the eye movement is shown in Figure 4C. There was no significant interaction between the main effects [F (3, 42) = 1.706, *p* = 0.180]. There was no significant main effect of forearm position [F (1, 14) = 1.184, *p* = 0.295] and task [F (3, 42) = 1.887, *p* = 0.146].

### 3.2. RT of EMG Response

**Figure 5:**
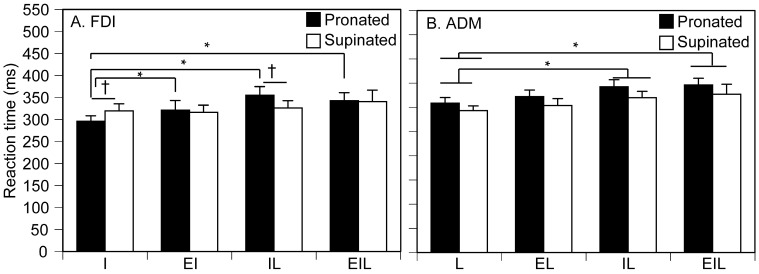
Reaction time of FDI and ADM. Data points indicate means and error bars indicate the stand error of the means. Each asterisk indicates the significant difference between the tasks (*p* < 0.05). Each dagger indicates a significant difference between forearm positions (*p* < 0.05).

The RT of the FDI-EMG response is shown in Figure 5A. There was a significant main effect of the task [F (1.911, 26.753) = 5.795, *p* = 0.009]. There was a significant interaction between the main effect of forearm position and that of the task [F (1.890, 26.464) = 4.146, *p* = 0.029]. The test of simple main effect revealed that the RT in the pronated forearm position was longer than that in the supinated forearm position in the IL task [F (1, 54) = 8.24, *p* = 0.006]. Another test of simple main effect revealed that the RT in the supinated forearm position was longer than that in the pronated forearm position during the I task [F (1, 54) = 5.469, *p* = 0.023]. There was a significant simple main effect of the task in the pronated forearm position [F (3, 84) = 8.843, *p* < 0.001]. The RT was significantly longer when the index finger movement was executed with little finger movement (IL task) or eye and little finger movements (EIL task) in the pronated forearm position (*p* < 0.001).

The RT of the ADM-EMG response is shown in Figure 5B. There was no significant interaction between the position and the task [F (1.773, 24.827) = 0.110, *p* = 0.874]. The RT in the pronated forearm position was significantly longer than that in the supinated forearm position [F (1, 14) = 6.505, *p* = 0.023]. There was a significant main effect of the task [F (3, 42) = 7.971, *p* < 0.001]. The RT in the IL task (*p* = 0.014) and EIL task (*p* = 0.007) was significantly longer than the L task.

### 3.3. EMG Amplitude

**Figure 6:**
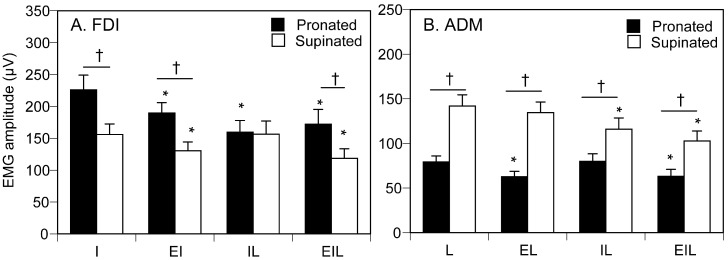
EMG amplitude of FDI and ADM. Data points indicate means and error bars indicate the stand error of the means. Each asterisk indicates a significant difference from I or L task (*p* < 0.05). Each dagger indicates a significant difference between forearm positions (*p* < 0.05). FDI, first dorsal interosseous muscle; ADM, abductor digiti minimi.

The EMG amplitude of FDI (FDI-EMG amplitude) is shown in Figure 6A. There was a significant interaction between forearm position and task [F (3, 42) = 6.348, *p* = 0.001]. A test of the simple main effect revealed that the EMG amplitude in the pronated forearm position was larger than that in the supinated forearm position in the EIL [F (1, 46) = 14.924, *p* < 0.001], EI [F (1, 46) = 18.287, *p* < 0.001], and I tasks [F (1, 46) = 25.666, *p* < 0.001]. There was a significant simple main effect of the task in the supinated forearm position [F (3, 84) = 4.775, *p* = 0.004]. The EMG amplitude in the EI or EIL tasks was smaller than that in the I task in the supinated forearm position (*p* < 0.05). There was a significant simple main effect of the task in the pronated forearm position [F (3, 84) = 11.180, *p* < 0.001]. The EMG amplitude of the EI, IL, or EIL task was significantly smaller than that of the I task in the pronated forearm position (*p* < 0.05).

The EMG amplitude of ADM (ADM-EMG amplitude) is shown in Figure 6B. There was a significant interaction between forearm position and task [F (3, 42) = 17.894, *p* < 0.05]. A test of simple main effect revealed that the EMG amplitude in the pronated forearm position was significantly larger than that in the supinated forearm position in the EIL [F (1, 20) = 20.424, *p* < 0.05], EL [F (1, 20) = 67.417, *p* < 0.05], IL [F (1, 20) = 17.053, *p* < 0.05], and L tasks [F (1, 20) = 50.927, *p* < 0.05]. There was a significant simple main effect of the task in the supinated forearm position [F (3, 84) = 19.763, *p* < 0.05]. The EMG amplitude of the IL or EIL task was smaller than that in the L task in the supinated forearm position (*p* < 0.05). There was a significant simple main effect of the task in the pronated forearm position [F (3, 84) = 5.834, *p* < 0.05]. The EMG amplitude in the EL or EIL task was smaller than that in the L task while the participants maintained the pronated forearm position (*p* < 0.05).

## 4. Discussion

### 4.1. Proprioceptive Influence

In the present study, forearm proprioception influenced the effect of the saccade eye movement on the finger muscle’s motor excitability or central motor drive to the finger muscle. On the one hand, there was no significant interaction between the task and forearm position, and no significant main effect of forearm position on ADM-RT. On the other hand, the task significantly interacted with forearm position for FDI-RT as well as for FDI- and ADM-EMG amplitude. These findings indicate the influence of forearm proprioception on the central motor drive to the independent finger, as well as on the motor excitability of the independent and dependent finger muscles, depends on the additional involvement of the central motor drive to the other finger or to the eyes.

**Figure 7:**
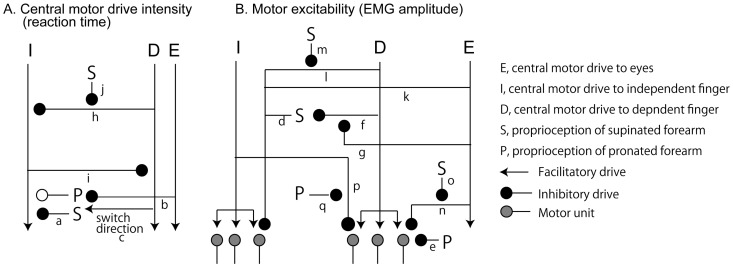
Model explaining the interaction among the eyes and the fingers based on the present finding. The assumed mechanism is discussed with citing labels of this figure in Discussion.

The present finding contrasts with a previous finding showing that forearm proprioception did not influence the saccade eye movement-induced change in the corticospinal excitability of the finger muscle at rest [11]. The key difference between the previous and present findings lies in whether the central motor drive to the target muscle was present. In the present study, the central motor drive was provided to the target muscle, whereas it was not in the previous study. This difference supports the idea that forearm proprioception particularly influences the central motor drive to the target muscle. The schematic illustration of how proprioception influences the central motor drive based on this view is shown in Figure 7.

On the one hand, FDI-RT significantly differed between forearm positions when the index finger movement (I task) or index and little finger movements (IL task) were performed. On the other hand, there was no significant difference in FDI-RT between forearm positions when the eye movement was additionally involved (i.e., EI and EIL tasks). The eye movement was not involved in the I and IL tasks, but it was involved in the EI and EIL tasks. Thus, the finding indicates that forearm proprioception influences the intensity of the central motor drive to the independent finger muscle (Figure 7a), but this influence is masked by the central motor drive to the eyes (Figure 7b).

The effect of forearm position on FDI-RT was opposite between the I and IL tasks: the RT in the I task was longer, whereas the RT in the IL task was shorter in the supinated position. The difference between the I and IL tasks is the involvement of the central motor drive to the little finger. Thus, the finding indicates that the direction of the proprioceptive influence on the central motor drive to the independent finger muscle is switched by the central motor drive to the little finger muscle (Figure 7c).

In both muscles, the simple main effect of forearm position on the EMG amplitude was found, except for FDI-EMG amplitude in the IL task. In the IL task, the little finger movement was additionally involved. Thus, the present finding indicates that forearm proprioception influences the motor excitability of the independent (Figure 7d) or dependent finger muscles (Figure 7e), but this influence on the independent finger muscle is masked by the central motor drive to the little finger muscle (Figure 7f). When the eye movement was added to the little finger movement (i.e., EIL), forearm proprioception influenced FDI-EMG amplitude. Taken together, masking of the proprioceptive influence on the motor excitability of the independent finger caused by the central motor drive to the little finger muscle (Figure 7f) is cancelled by the central motor drive to the eyes (Figure 7g).

The proprioceptive influence on the motor units reaching the threshold by the central motor drive to the finger (i.e., motor excitability at the movement onset) was opposite between the two muscles: FDI-EMG amplitude was greater, whereas ADM-EMG amplitude was smaller in the pronated forearm position. FDI is the primary mover for index finger abduction, while ADM is the primary mover for little finger abduction. The directions of these movements are opposite along the medial-lateral axis. When the forearm is pronated, the little finger abduction is directed to the right. In contrast, the index finger abduction is directed to the left when the forearm is pronated. The eyes moved to the right. Therefore, one might speculate that the consistency in the direction of eye and finger movements underlies the opposite effects observed. However, the opposite tendency persisted even when the eye movement was absent (e.g., during the I vs. L task). Thus, the consistency of eye and finger movement directions cannot account for the opposite effects observed. The most likely explanation for the present findings is that the central motor drive to the finger muscle is typically enhanced when a finger of the right hand moves to the right.

The influence of forearm proprioception on the interaction between the fingers and between the eyes and finger was different between the independent and dependent fingers. The independent finger plays a key role in the precise manipulation of objects, such as during pinching. In contrast, the dependent finger does not contribute to such precision tasks but contributes to power grip. Accordingly, the finger-dependent proprioceptive influence on the interaction between the fingers may be related to the different functional roles.

### 4.2. Central Motor Drive to Finger

The simple RT in the present study represents the time required to reach the excitability of the motor units to the threshold caused by the central motor drive, and thus, reflects the intensity of the central motor drive. Figure 7A illustrates the model, derived from simple RT results, that describes how the central motor drive to one muscle affects the intensity of the drive to another. First, in the pronated forearm position, FDI-RT was significantly longer when either the little finger movement (IL task) or the little finger and eye movements (EIL task) were additionally involved. Second, regardless of forearm position, ADM-RT was significantly longer when the index finger movement (IL task) or the index and eye movements (EIL task) were additionally involved. The commonly added movement causing the increase in the RT was the movement of the finger other than the examined one (i.e., little finger movement for FDI-RT and index finger movement for ADM-RT). Thus, the present finding indicates that the central motor drive to one finger muscle is suppressed by the central motor drive to the other finger muscle. This suggests a mutual inhibitory interaction between the central motor drives to the independent and dependent finger muscles (Figure 7h,i).

When the forearm was pronated, the central motor drive to the little finger increased FDI-RT. However, this significant increase was absent when the forearm was supinated. This can be explained by the notion that the proprioception of the supinated forearm suppresses the inhibitory influence of the dependent finger central motor drive on the central motor drive to the independent finger muscle (Figure 7j). Such an effect was absent in ADM-RT; the increase in ADM-RT caused by the additional involvement of the index finger movement was observed regardless of forearm positions. This suggests that there is no proprioceptive modulation of the inhibitory drive from the independent finger’s central motor drive to that of the dependent finger muscle.

### 4.3. Motor Excitability

In the present study, the EMG amplitude at the movement onset was measured. The EMG amplitude represents the motor units activated by the central motor drive to the target finger. In other words, the EMG amplitude is the indicator of the motor excitability. Figure 7B presents a model illustrating how the central motor command affects motor excitability at movement onset. On the one hand, in the pronated forearm position, FDI-EMG amplitude was decreased either by additional involvement of the little finger (IL task) or eye movement (EI task). This indicates that the central motor drive either to the eye (Figure 7k) or dependent finger (Figure 7l) suppresses the motor excitability of the independent finger muscle while the proprioception of the pronated forearm is provided. On the other hand, FDI-EMG amplitude in the supinated forearm position was decreased by the additional involvement of the eye movement (EI task) or eye and little finger movements (EIL task). The common additional involvement, in which the significant influence was observed, was the eye movement. Thus, the central motor drive to the eyes suppresses the motor excitability of the independent finger muscle regardless of the proprioceptive influence of forearm position (Figure 7k). Moreover, the central motor drive to the eyes suppresses the motor excitability of the independent finger muscle, but that to the dependent finger does not while the proprioception of the supinated forearm is provided. Accordingly, the central motor drive to the dependent finger suppresses the motor excitability of the independent finger muscle (Figure 7l), but this effect is masked with the proprioception of the supinated forearm (Figure 7m).

In the pronated forearm position, ADM-EMG amplitude was significantly smaller when the eye movement (EL task) or the index finger and eye movements (EIL task) were additionally involved. In both tasks, the additional movement was consistently the eye movement. These findings indicate that the motor excitability of the dependent finger muscle was suppressed by the central motor drive to the eyes (Figure 7n), but this influence was masked by supinated forearm proprioception (Figure 7o). In the supinated forearm position, ADM-EMG amplitude was significantly smaller when the index finger movement (IL task) or the eye and index finger movements (EIL task) were additionally involved. In these tasks, the additional movement was consistently the index finger movement. This indicates that the motor excitability of the dependent finger muscle was suppressed by the central motor drive to the index finger muscle (Figure 7p); however, this influence was masked by pronated forearm proprioception (Figure 7q).

### 4.4. Central Motor Drive to Eyes

In the present study, the central motor drive to the eyes suppressed the motor excitability of the finger muscles. In a previous study, smooth pursuit eye movement in the pronated forearm position suppressed the motor evoked potentials in FDI, ADM, and flexor carpi radialis muscle at rest [9]. This finding has been explained by the view that the inhibitory drive to the finger is provided to maintain it at rest against the central motor drive to the eyes that enhances the corticospinal excitability of the finger muscle. However, this previous finding is not comparable to the present finding, as those findings are regarding the effect of the smooth pursuit eye movement. The modulation of the corticospinal excitability of the finger muscle at rest induced by the saccade eye movement was absent in a previous study; the saccade eye movement with visual occlusion suppressed the corticospinal excitability of FDI, APB, and ADM at rest regardless of forearm position [11]. This study investigated the effect of the saccade eye movement, which was the same as the present study, but the inhibitory drive to the finger caused by the eye movement was observed while the fingers were at rest. In the present study, the inhibitory drive to the finger caused by the eye movement was found even when the finger was moving; i.e., the eye movement decreased the EMG amplitude of the finger muscles at the movement onset. Thus, the inhibitory mechanism underlying the present finding cannot be attributed to the mechanism underlying the process to maintain the finger at rest.

### 4.5. Influence of Finger on Eyes

In the present study, there was no significant effect of the finger motor drive on the motor excitability of the eyes. The motor plan for a saccade eye movement to the next target was suppressed during moving the hand, but not when the hand was at rest [12]. In the present study, the hand remained at rest during the RT. Thus, the present finding is consistent with this previous finding that the saccade eye motor plan is not suppressed while the hand is not moving (i.e., before the movement onset).

### 4.6. Between Fingers Interaction

The present study demonstrated the mutual inhibitory interaction between the independent and dependent fingers. Several previous studies have also reported the mutual inhibitory interaction between the fingers. Additional involvement of the index finger abduction increased ADM-RT, or that of the little finger abduction increased FDI-RT [13]. During the index finger abduction, ADM-MEP remained unchanged, despite the increase in the F-wave persistence and background EMG [49], suggesting that the central motor drive to FDI suppresses the primary motor cortex of ADM. FDI-MEP during the tonic contraction was suppressed by ADM contraction [14,15]. In contrast, ADM-MEP during the tonic contraction was not suppressed by FDI contraction [15]. Force production by one finger decreases force production of the other fingers, whereas cessation of force production by the same finger increases that [16]. Such a mutual inhibitory mechanism may be related to the surround inhibition system, in which the motor execution of the target suppresses the adjacent motor system to focus on the target [21].

### 4.7. Limitations

Based on the task instruction, the participants may have altered the strategy regarding the motion velocity and direction. However, these variables were not measured in the present study. Thus, we cannot rule out a possible influence of the movement direction or amplitude on the present finding. In addition, we used raw EMG amplitude. Raw EMG signals are sensitive to factors such as anatomical and physiological characteristics, electrode placement, and skin impedance levels [50]. This may have increased the inter-participant variability of the EMG amplitude, causing an increase in type II error for the results of the ANOVA. A review article indicated that using the raw EMG is not suitable for group comparisons [51]. However, in the present study, a comparison was made within each participant using repeated measures ANOVA. Thus, the use of raw EMG amplitude remained acceptable.

### 4.8. Future Direction

In the present study, the motor task involved a discrete and phasic motor response. However, in the real world, humans often move their eyes and fingers continuously to track moving targets. It remains unclear whether the present finding regarding the three-way interaction among the central motor drive to the eyes and fingers is applicable to the continuous manual tracking task. The continuous manual tracking task involves online movement feedback. Thus, the underlying control mechanism may differ from that involved in discrete tasks. Indeed, a previous study reported that continuous tasks involve unique motor control and sensory integration [52]. Accordingly, in the future, it would be important to investigate whether the observed motor drive interactions persist during continuous manual tracking tasks. Investigating such a task would help deepen understanding of eye–hand coordination.

## 5. Conclusions

The central motor drive to the finger does not influence the eye motor excitability and central motor drive to the eyes. The central motor drive to a finger suppresses the other finger’s motor excitability or the central motor drive to the other finger muscle. Forearm proprioception interacts with the mutual inhibition between the fingers or between the finger and eyes. The central motor drive to the eyes suppresses the motor excitability of the independent finger muscle but does not influence the central motor drive to either the independent or the dependent finger muscles.

## Data Availability

Data are unavailable due to privacy restriction.
